# An Individual Patient Data Meta-Analysis with Colombian Studies on the Effect of Dark Chocolate Consumption on Cardiovascular Risk Parameters

**DOI:** 10.1155/2020/3419598

**Published:** 2020-12-05

**Authors:** Leidy Alvarez, Javier Contreras, Mónica Giraldo

**Affiliations:** ^1^Group of Clinical Epidemiology, University of Antioquia, Medellín 05001, Colombia; ^2^Department of Pediatrician, Group of Clinical Epidemiology, University of Antioquia, Medellín 05001, Colombia; ^3^Department of Microbiology and Parasitology, Group of Primary Immunodeficiencies, University of Antioquia, Medellín 05001, Colombia

## Abstract

**Background:**

It is postulated that cocoa solids possess cardioprotective capacity by various mechanisms. In the different cocoa studies evaluating cardiovascular disease, there are no conclusive data on the role it plays in controlling the lipid profile and anthropometric variables, perhaps because the concentration of cocoa, the geographical origin of the population, and the different concentrations supplied lead to a high heterogeneity of results. This study aims to estimate the effect of consuming cocoa-rich chocolate compared to placebo on the lipid profile and anthropometric variables based on data from three clinical trials conducted in Colombia.

**Methods:**

Meta-analysis of individual data from three randomized clinical trials conducted in Colombia. The entire population of the primary studies was included, which was reassigned into intervention groups if they consumed 50 grams of 70% concentrated cocoa or placebo, which was considered to be cocoa-free or with a concentration less than 50 grams. The variables at the beginning of the study were analyzed with medians, interquartile ranges, means, and deviations according to whether they met the normality assumption. Multiple imputations were used to manage missing data and were analyzed using the two approaches proposed for this type of study, that of one and two stages. In the two-stage approach, the data were weighted on a conventional Forrest plot, while in the one-stage approach, linear regressions with mixed models were applied. This study is governed by the regulations described in the 2013 Declaration of Helsinki and by article 11 of Resolution 8430 of 1993, which classifies it as a risk-free study.

**Results:**

A total of 275 participants were included, who consumed cocoa or placebo for 81 days on average; 52.7% were female and few smoked at the time of the intervention (31/275). Physical activity performed in number of hours per week was comparable between the intervention groups. When evaluating total cholesterol, low-density cholesterol (LDL), high-density cholesterol (HDL), triglycerides, abdominal circumference, and final body mass index with both the one-stage and two-stage approaches, there were no significant differences between the two groups.

**Conclusions:**

According to the results obtained in the meta-analysis, the consumption of cocoa in the Colombian population does not seem to significantly modify variables such as lipid profile, abdominal circumference, and body mass index. This conclusion according to the quality of the evidence has a weak recommendation and a low-to-moderate certainty. However, the analysis through the two proposed approaches yielded similar results.

## 1. Introduction

The complications derived from hypertension, diabetes mellitus, overweight, and obesity represent an important cause in morbidity and mortality, which translates into a high demand for the use of services and a high cost in the health of the population. The average cost of medical services caused by a cardiovascular patient in Colombia calculated between 2002 and 2011 was approximately 12.8 million pesos and exceeded by nearly 50% the costs generated by a patient with neoplastic disease [1].

It is estimated that 80% of these complications can be prevented by adopting healthy lifestyle habits, such as a balanced diet, regular exercise, and cigarette abstinence [2]. There is evidence that the modification of the lipid profile and the anthropometric variables represents a benefit in reducing the development of cardiovascular disease (CVD) [3]. Most of the clinical trials that evaluate weight reduction and improvement in lipid profile agree that adherence to a healthy diet is important for the control of these risk factors [4], so adding food products with high acceptability that modifies these risks in people could improve adherence, which would be reflected in the long term in the reduction of cardiovascular events.

Chocolate is a widely available cocoa-based product. It is estimated that in the world 19.8 pounds per capita are consumed on average per year, with Germany at the top of the list [5]. It is postulated that cocoa solids possess cardioprotective capacity by several mechanisms, among which it can be pointed out: decreased endothelial inflammatory response induced by atheromatous plaque, antioxidant capacity, increased vasodilation, decreased platelet reactivity, reduced blood sugar index, and insulin resistance [6–9]. Therefore, adding cocoa to the usual eating plan together with the other nutritional and physical activity recommendations could be an effective strategy to control CVD risk factors [9, 10].

In the different cocoa studies, there are no conclusive data for the control of the lipid profile and anthropometric variables due to the high heterogeneity of the results. This heterogeneity may be due to the fact that the amount of cocoa components present in the body after ingestion will depend on the intake levels, on individual factors such as genetic polymorphisms of enzymes that metabolize flavonols (main components of cocoa), from the presentation in which cocoa is administered, plasma concentration, processing techniques, the origin of the person, among others [11].

Currently, studies evaluating the impact of cocoa on metabolism and hypertension are controversial. The differences in the beneficial effect of cocoa in the diet may be due, among other variables, to the genetic characteristics of each population and their habits [12]. Several studies in in vivo models [13, 14] show how, according to the form and frequency of cocoa consumption, it can change the intestinal flora and modify not only the genes associated with its metabolization, but also the genes associated with the endocrine system and endothelial tissue. These changes can be genetically transmitted, generating populations with a response to genetically conditioned cocoa that may differ even in geographically close places such as different cities in the same country [15, 16]. Therefore, when evaluating the impact of cocoa on the lipid profile, it is necessary to know and, if possible, control these variables, which is possible if we analyze the impact of cocoa in populations where these characteristics are similar.

Currently, in Colombia, we have some studies with cocoa in the hypertensive population, where there was no reduction in blood pressure in the intervention group in contrast to what was published with other authors [17–20]; however, this may be related to the low power obtained with the sample size.

The objective of this study was to estimate the effect of chocolate consumption with a minimum of 70% cocoa solids compared to placebo on the lipid profile and anthropometric variables of a population formed from three clinical trials conducted in Colombia. In the meta-analysis, the three included studies belong to the same population, which has been previously studied and from the genetic and cultural point of view, the subjects that comprise it have little heterogeneity [21], which allows controlling, at least partially, the impact of these variables and evaluating the outcomes of interest by reducing the variability that different populations with different dietary habits and genetic patterns could generate.

## 2. Materials and Methods

The individual data meta-analysis methodology was used, which, unlike the conventional aggregate data meta-analysis methodology, consists of a quantitative synthesis of individual data derived from the participants of different studies.

For this study, 3 randomized controlled clinical trials carried out in the city of Medellín were chosen as the primary source. Individual data from the entire population were requested and reassigned into groups to meet the definition of intervention and comparator for this study:

Intervention: participants who have consumed chocolate bar presentations with a concentration greater than or equal to 50 g with 70% cocoa solids or 200 mg of polyphenols during a follow-up of at least 4 weeks.

Comparator: participants who have consumed chocolate free of cocoa solids or presentations of white chocolate bar or with less than 50 g. During a follow-up of at least 4 weeks, this period was chosen because it has been seen in the subgroup analyses of the systematic reviews, that during this follow-up it has been possible to show changes in the lipid profile and anthropometric variables [17].

This study, in accordance with the question posed, only included the Colombian population to control variables such as genetic ancestry, so there was no systematic review prior to the meta-analysis. In this sense, only the information extracted as important for this study from the databases provided by the main authors of the randomized clinical trials is included.

### 2.1. Study Selection

Three randomized clinical trials conducted with native Colombians were included. Among the characteristics of each of them, the following can be highlighted:

  Giraldo et al. [19]: this parallel clinical trial aimed to evaluate the effect of cocoa consumption compared to white chocolate on blood pressure and cardiovascular and immunological parameters, in mestizo individuals with a de novo diagnosis of arterial hypertension Stage 1 essential. Male and female volunteers aged 18 to 65 years with no history of CVD, not obese, or smokers with a diagnosis of stage 1 hypertension (systolic blood pressure between 140 and 159 mm/Hg and/or diastolic blood pressure) were chosen between 90 and 99 mm/Hg). At the moment without treatment with antihypertensive or antiplatelet drugs, a total of 69 participants were randomized and 66 were analyzed (34 in the control group and 32 in the placebo group). Each individual was in the study 84 days. The intervention group consumed 50 g of chocolate with 70% cocoa solids daily and the placebo group chocolate free of cocoa solids.
  Giraldo et al., 2013 [20]: this second clinical trial aimed to estimate the effect of different doses of cocoa, compared to placebo, in the reduction of diastolic blood pressure. Male and female volunteers between the ages of 18 and 65 years with no history of CVD, not obese, or smokers with a diagnosis of stage 1 or 2 hypertension, treated with antihypertensive drugs were chosen. 125 participants were randomized and 124 were analyzed. Individuals in the intervention group were assigned to 4 treatment arms with 6.5 g, 12 g, 25 g, and 50 g of chocolate with 70% cocoa solids while the placebo group received 50 g of chocolate free of cocoa solids, for an individual duration of 18 weeks.
  Giraldo et al. [9]: the objective of this third clinical trial was to determine the effect of cocoa consumption in patients with metabolic syndrome. For this, nondiabetic male and female volunteers aged 18 to 70 years with insulin resistance were chosen. Pregnant women and people who reported a consumption of chocolate greater than 50 g per week were excluded. 80 participants were randomized and 75 were analyzed (37 in the control group and 38 in the placebo). The intervention group had to consume a total of 50 g of chocolate with 70% cocoa solids and in the placebo group, cocoa solids-free chocolate, for an individual duration of 8 weeks.

### 2.2. Risk of Bias in Individual Studies

Primary risk assessment of bias was assessed by an outsider using the Cochrane Collaboration tool, which includes sequence generation, allocation sequence concealment, blinding, incomplete outcome data, selective reporting of the results, and other possible sources of bias [22].

### 2.3. Types of Outcome Measures

The mean differences between the groups at the end of the intervention were estimated on the following cardiovascular risk indicators: total cholesterol (TC), triglycerides (TG), high-density cholesterol (HDL-C), low-density cholesterol ( LDL-C), body mass index (BMI), and abdominal circumference.

### 2.4. Collection Process and Analysis Plan

For the collection process, the databases were obtained in Access format. Relationship trees were created to extract important data for this new study. The extracted data was transferred to the SPSS format.

After obtaining the results of the three clinical trials, an information extraction was carried out in each of them (see Annex 1), in order to form three individual databases and with them a joint database.

The description of the population was made by reporting frequencies and percentages for categorical variables, median with interquartile ranges for continuous variables that did not meet the criteria of normality with the Kolmogorov–Smirnov test and mean with standard deviation for those that did. They fulfilled that assumption.

An analysis of lost data was carried out in the variables defined as descriptive outcomes and with the Little MCAR streak test; this in order to evaluate the possibility of making multiple imputations fulfilled the assumption of completely random losses in each of the databases [23]. Once the information was obtained, the outcomes were analyzed using the two approaches for managing individual data (the one- and two-stage) [24]; this is in order to observe how different the results are when applying one approach or another.

For the two-stage approach in step one, the participants were reassigned in order to comply with the definition previously proposed; the means and their respective standard deviation were calculated, taking into account the compliance with normality of the variables. Once these data were obtained, we proceeded to the second step, where effect estimates between studies were combined in a meta-analysis using the generic inverse variance technique in a random effects model. The *I*^2^ statistic was used to calculate heterogeneity, and funnel plot publication bias was evaluated.

For one-stage, data were analyzed in the pooled database, where each trial was identified with an independent variable. As the outcomes were continuous, the analyses were made using a mixed effects linear regression model, taking into account the origin of the participants in each of the studies. The independent variable taken into account for the regression corresponded to the intervention, where the null value is related to the placebo and the unit to the administration of cocoa.

The alpha value defined as the cut-off point was 0.05. All analyses were performed with SPSS version 23 and Review Manager (v5.3, the Cochrane Collaboration) software. To assess the quality of the evidence, the GRADE guidelines were used using the statistical software GRADEpro (the Cochrane Collaboration).

### 2.5. Ethical Considerations

This study is governed by the regulations described in the 2013 Declaration of Helsinki and by article 11 of Resolution 8430 of 1993 from Colombia, which classifies it as a without risk.

In the primary studies, we verified the endorsement of the ethics committee, and techniques of concealment of participant identification data were performed.

### 2.6. Protocol Registration

The protocol of this study has not been published in databases or institutional pages.

## 3. Results and Discussion

### 3.1. Risk of Bias of Included Studies

In general, for all the included studies, the criteria of random sequence generation were met (statistical software was used for all three cases), allocation concealment (with the properly sealed envelopes method), masking of the intervention and outcomes, and selective reporting of data. Some of the evidenced biases were related to the nonevaluation of the data of patients in whom follow-up was lost and to an incomplete sample size in two of the studies.

**Figure 1:**
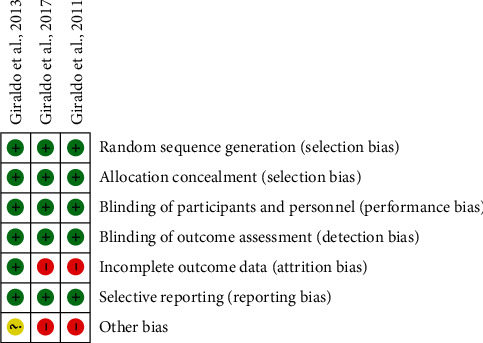
Traffic light of risk of bias. Each color represents the presence and absence of the bias presented in the last column.

Figure 1 shows the traffic light of risk of bias.

### 3.2. Baseline Characteristics of the Participants

Table 1 shows the general baseline characteristics and by treatment group of the included population. With an average duration of 81 days (minimum 32, maximum 126 days), a total of 275 participants were analyzed for the three studies, which were mostly female, with a similar percentage per group (53.5% vs. 52.3%). None of the participants had a personal history of ischemic disease or diabetes; the median hours of physical activity were similar in the two groups, with a median of 2 hours, as well as the median age with a value of 51 years for the total population. More people smoked in the placebo group (11 vs. 20 cigarettes per day); however, the median number of cigarettes smoked was similar in both groups. Regarding the basic characteristics of the outcomes of interest, the means were similar in both groups.

### 3.3. Handling of Lost Data

The variables defined as outcomes were descriptively evaluated in each of the databases, observing the frequency and pattern of losses (see Annex 2). In no case was a pattern similar to nonrandom loss evident. When applying Little's MCAR streak test, it can be observed that in all cases the assumption of completely random losses was fulfilled, for not rejecting the null hypothesis with a *p* value for the test less than the defined alpha level, as observed in Table 2.

With these data, multiple imputation was carried out with a total of 10 iterations and applying an automatic model, which could apply a completely conditional specification (Monte Carlo method and Markov chains) or a monotonic method, taking into account the type of losses [23, 25].

## 4. Synthesis of Results with the Two-Stage Approach

**Figure 2:**
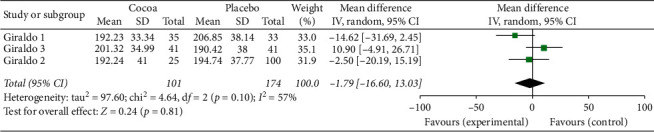
Meta-analysis of the effect of cocoa consumption on total cholesterol (TC) as compared with placebo. The sizes of the data markers indicate the weight of each study in the analysis. IV: inverse variance.

**Figure 3:**
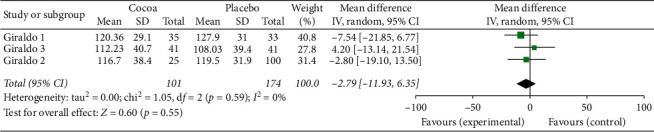
Meta-analysis of the effect of cocoa consumption on LDL cholesterol as compared with placebo. The sizes of the data markers indicate the weight of each study in the analysis. IV: inverse variance.

**Figure 4:**
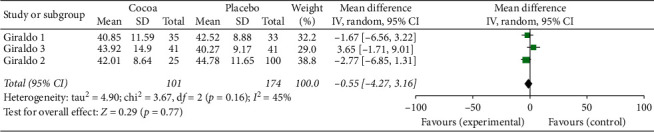
Meta-analysis of the effect of cocoa consumption on HDL cholesterol as compared with placebo. The sizes of the data markers indicate the weight of each study in the analysis. IV: inverse variance.

**Figure 5:**
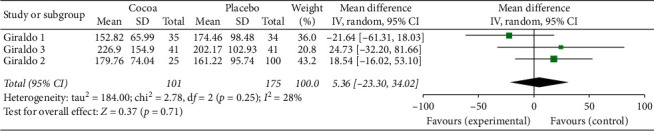
Meta-analysis of the effect of cocoa consumption on triglycerides as compared with placebo. The sizes of the data markers indicate the weight of each study in the analysis. IV: inverse variance.

**Figure 6:**
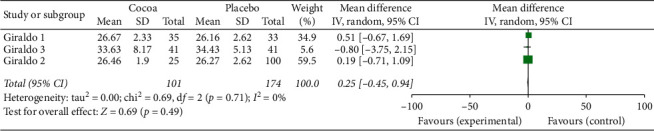
Meta-analysis of the effect of cocoa consumption on body mass index (BMI) as compared with placebo. The sizes of the data markers indicate the weight of each study in the analysis. IV: inverse variance.

**Figure 7:**
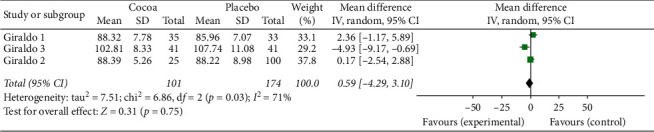
Meta-analysis of the effect of cocoa consumption on abdominal waist as compared with placebo. The sizes of the data markers indicate the weight of each study in the analysis. IV: inverse variance.

For this first two-stage analysis, the three studies were independently managed. We can see from Figures 2–7 that, for the majority of cases, except for the body mass index and triglycerides, the administration of cocoa reduces the means of these values (in the weighted analyses there are absolute values of reduction of −2.79 at −0.55 mg/dl); however, this reduction was not significant and cannot be considered clinically important for any of the variables.

The highest percentage of heterogeneity in these results was achieved in the abdominal waist outcome (I2: 71%) and the lowest in LDL cholesterol and body mass index (BMI), which was 0%.

## 5. Synthesis of Results with a One-Stage Approach

When analyzing the data in each of the outcomes, we can see results similar to those observed with the two-stage approach. While it is possible to see reductions in the group that consumed cocoa, these are not significant.  Table 3 shows the result of each mixed effects linear regression.

When controlling for variables that had been considered as sources of heterogeneity, due to the hypothesis that the effect of polyphenol compounds lasts longer in blood due to variables such as the ancestral origin of the population, changes in polymorphisms and the genetic variance of the cocoa absorption and response systems, administered cocoa concentration in in vivo studies, the placebo used, and the duration of the intervention, our analyses show that there is no statistically or clinically significant change in total cholesterol, LDL-C, HDL-C, triglycerides, body mass index, and abdominal circumference after the administration of 50 grams of 70% cocoa chocolate in the Colombian population for at least 4 weeks. These results were obtained using the meta-analysis methodology of individual data, which allowed us to control for factors that were considered sources of heterogeneity in previous studies, expand the sample size, regroup participants, and work with missing data.

For the analysis of the data, the one- and two-stage approaches recommended in the literature were used; in both cases, similar results were obtained when considering the possible sources of discrepancies described [24], probably related to this, with the use of controlled clinical trials. As a primary source, they allow not having to adjust for certain variables that could be confusing and for the corrections that were applied to avoid them. These corrections are related to the choice of a sample size greater than 30 subjects, which allows considering the data with normal distribution in both approaches (central limit theorem), the choice of the random intercept in the one-step method in mixed regression, the weighting of the results, in the two-stage approach, using the Mantel and Haenszel method (due to the existence of possible unbalanced data) and finally considering random effects, in both cases, for the parameters of interest. It should be borne in mind that possible interactions were not evaluated in this study, where the one-stage models are recommended [26].

When talking about cocoa and lipid profile, Grassi et al. [27] reported that dark chocolate was associated with a decrease in total cholesterol (−6.5%; *p* < 0.0001) and LDL-C (−7.5%; *p* < 0.0001). No effect of dark chocolate on HDL-C or triglyceride (TG) concentrations was observed. In the meta-analyses of Tokede et al. [18] and Jia et al. [3], where populations from all continents were included, with an emphasis on European and Asian, there were no significant differences in HDL-C or triglycerides, such as what occurred in this study; although a difference of 5.9 mg·dl (95% CI: −10.47, −1.32) in LDL cholesterol was evident in the Tokede meta-analysis, the heterogeneity of this result is much greater than that obtained (32% vs. 0%); this is probably related to the quality of the included studies, the duration of the intervention (from 2 to 12 weeks), and the origin of the population.

Regarding the body mass index and the abdominal waist, Kord et al. also in a systematic review of cocoa studies in populations from different continents [17] showed that there was no clinically important change in these variables; however, their results were heterogeneous when evaluating the population with obesity, doses of more than 30 grams of cocoa were administered, and duration of intervention was greater than 4 weeks, where it seemed that a clinical benefit was obtained. In our case, even controlling the dose, presentation, and duration of administration, the differences do not report significant results.

Another clinical trial type study conducted in Iran analyzed the effects of six weeks of dark chocolate supplements combined with jump rope exercises on inflammatory cytokines, adipokines, and body composition in 48 obese adolescent males, showing that among the groups of patients who received dark chocolate versus placebo, no significant differences were observed between the BMI, as in our study [28].

One of the major limitations of this study is related to the absence of a systematic review and the inclusion of only 3 clinical trials for the analyses, which did not have the main objective of evaluating the changes in the evaluated variables. This was due in part to the resource constraint for study funding, and the willingness to submit individual data by the lead investigators at the time set for this purpose. Probably, this limitation has caused a possible publication bias, trying to correct with the choice of studies in Colombian individuals, which have the advantage of controlling heterogeneity sources such as the genetic characteristics, composition, and processing of cocoa. With the funnel charts represented in Annex 3, as there are few studies, it is difficult to see the distribution of the analyzed studies; however, in all cases, it seems that with the included studies, a representative sample of a population could be obtained that, in this case, would be the Colombian one. Another limitation was related to the fact that, although they tried to manage possible modifying variables, it was not possible to carry out subgroup analyses due to the type of baseline risk of each of the participants, such as the presence of hypertension or dyslipidemia, or analysis of sensitivity, since with the sample size it was not possible to do it properly.

## 6. Conclusions

In conclusion, despite the limitations described, the administration of cocoa does not seem to positively modify the outcomes studied. In addition, these results show that population characteristics can have an impact on studies, so it is important that health decisions are made with data specific to each population that are aimed at making recommendations against or in favor, as is our case. We also recommend that future research with cocoa in our population look for direct outcomes, such as the development of cardiovascular disease, which although in the literature there are mainly observational studies, it seems that in other populations studied such as the European consumption of a minimum of 45 grams could reduce the risk of development of cerebrovascular disease, heart failure, and myocardial infarction [29].

## Figures and Tables

**Table 1:** Characteristics of the study population, presented according to the distribution of the variable.

	Total (275)	Cocoa (101)	Placebo (174)
Female, *n* (%)	145 (52.7)	54 (53.5)	91 (52.3)
Age, years, median (RIQ)	51.4 (43.3–58.1)	50.4 (40.7–58)	51.6 (44–58.3)
Scholarship, years, median (RIQ)	13 [9–16]	13 [8–17]	13 [9–16]
Smoke, *n* (%)	31 (11.3)	11 (10.9)	20 (11.49)
No. of cigarettes, median (RIQ)	0 (0–10)	0 (0–98)	0 (0–5)
Physical activity, *n* (%)	180 (65)	67 (66.3)	113 (65)
Hours of physical activity per week, median (RIQ)	2 (0–4)	2 (0–4)	2 (0–4)
History of hypercholesterolemia, *n* (%)	116 (42)	41 (40.6)	75 (43.1)
BMI^∗^, mean (RIQ)	27.6 (25.7–30.7)	28.24 (26–31.3)	27.5 (25.1–30.6)
TC^∗∗^, mean ± (SD)	194.08 (38.5)	192.5 (33.7)	195.02 (41)
LDLC^∗∗∗^, mean ± (SD)	117.61 (34.76)	115.5 (33.3)	118.81 (35.6)
HDL-C^§^, mean ± (SD)	41.9 (12.11)	40.67 [11]	42.6 (12.7)
TG^§§^, median (RIQ)	150 (115–220.5)	161 (124.5–230.5)	144 (108–207)
Abdominal waist, median (RIQ)	94 (87.22–102.6)	96 (89–104.5)	93 (87–100)

^∗^BMI: body mass index (Kg/cm^2^); ^∗∗^CT: cholesterol total (mg/dL); ^∗∗∗^LDL-C: low-density cholesterol (mg/dL); ^§^HDL-C: high-density cholesterol (mg/dL); ^§§^TGS: triglycerides (mg/dL); RIQ: interquartile range; SD: standard deviation.

**Table 2:** Streak test, a value of *p* greater than 0.05 indicates that the losses are not random.

	MCAR *chi squared*	*p*
Cocoa 1	14.19	0.77
Cocoa 2	37.06	0.094
Cocoa 3	7.64	0.95

**Table 3:** Result with a one-stage approach showing the intercept and the beta values calculated with the variable cocoa consumption.

Outcome	Intercept	Beta	95% CI
Final intragroup total cholesterol	196.37	−1.24	(−10.35; 7.89)
Final intragroup LDL cholesterol	118.67	−3.2	(−11.97; 5.56)
Final intragroup HDL cholesterol	43.05	−1.22	(−4.06; 1.61)
Final intragroup triglycerides	178.16	16.16	(−10.7; 43.03)
Final intragroup BMI	28.97	−0.206	(−1.3; 0.89)
Final intragroup abdominal waist	94.08	−1.17	(−3.42; 1.08)

## Data Availability

Data are available from the corresponding author upon request.

## Additional Points

*Recommendation GRADE* [30]. We can say with a very low-to-moderate certainty in modifying these outcomes that the recommendation to give 50 grams of 70% cocoa for a minimum period of 4 weeks is weak in terms of protection measures for the outcomes studied (see Annex 4).

## Supplementary Materials

Supplementary Materials In supplementary materials are described in detail as annex the list of variables, analysis of missing data, funnel plot, and GRADE table.
