# Supplementary material for: An Individual Patient Data Meta-Analysis with Colombian Studies on the Effect of Dark Chocolate Consumption on Cardiovascular Risk Parameters
Source: J Nutr Metab. 2020 Dec 5;2020:3419598. doi: 10.1155/2020/3419598 (PMC7739050; doi:10.1155/2020/3419598)
Supplement: Supplementary Materials — In supplementary materials are described in detail as annex the list of variables, analysis of missing data, funnel plot, and GRADE table. [file 3419598.f1.docx]

## **Supplementary Materials**

**Annex 1.** List of variables requested from the authors

| **Variable** | **Measurement level** | **Measurement scale** | **Measurement** |
| --- | --- | --- | --- |
| Sexo | Nominal qualitative | Female  Male | At the start of the study |
| Age | Nominal qualitative | According to years completed | At the start of the study |
| Scholarship | Nominal qualitative | Total number of years of study | At the start of the study |
| Hypertension | Nominal qualitative | Yes or No | At the start of the study |
| Glucose intolerance / insulin resistance / prediabetes | Nominal qualitative | Yes or No | At the start of the study |
| Hypercholesterolemia | Nominal qualitative | Yes or No | At the start of the study |
| Obesity or overweight | Nominal qualitative | Yes or No | At the start of the study |
| History of ischemic disease | Nominal qualitative | Yes or No | At the start of the study |
| Physical activity | Discrete quantitative | Number of hours per week | At the start of the study |
| Smoke | Discrete quantitative | Daily number of cigarettes | At the start of the study |
| Weight | Continuous quantitative | kilograms | Result at the beginning and end of the study |
| Size | Continuous quantitative | meter | Result at the beginning and end of the study |
| Abdominal waist | Continuous quantitative | centimeters | Result at the beginning and end of the study |
| Total cholesterol | Continuous quantitative | milligrams per deciliter (mg/dL) | Result at the beginning and end of the study |
| HDL- C | Continuous quantitative | milligrams per deciliter (mg/dL) | Result at the beginning and end of the study |
| LDL - C | Continuous quantitative | milligrams per deciliter (mg/dL) | Result at the beginning and end of the study |
| Triglycerides | Continuous quantitative | milligrams per deciliter (mg/dL) | Result at the beginning and end of the study |
| Cocoa quantity administered | Continuous quantitative |  | Throughout the study |
| Duration of the intervention | Continuous quantitative | Days |  |
| Managed presentation | Nominal qualitative | Liquid, Solid, In bars |  |
| Comparator type | Nominal qualitative | Comparator name |  |
| Comparator Composition |  |  |  |
| Duration of the intervention with the comparator |  |  |  |
| Calories contributed from the comparator |  |  |  |

**Annex 2.** Analysis of missing data patterns with their graphical distribution and statistics


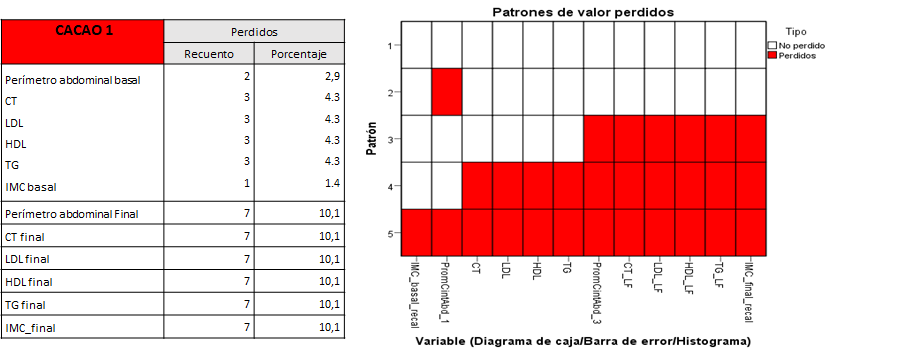


BMI

TGS

No missing

Missing

PATTERN

| **CACAO 1** | **Missing values** | |
| --- | --- | --- |
|  | **N** | **%** |
| Abdominal waist | 7 | 10.1 |
| Total cholesterol | 7 | 10.1 |
| HDL cholesterol | 7 | 10.1 |
| LDL cholesterol | 7 | 10.1 |
| Triglycerides (TGS) | 7 | 10.1 |
| Body mass index (BMI) | 7 | 10.1 |

ABD. WAIST

HDL-C

LDL-C

CT

| **CACAO 2** | **Missing values** | |
| --- | --- | --- |
|  | **N** | **%** |
| Abdominal waist | 5 | 4 |
| Total cholesterol | 4 | 3.2 |
| HDL cholesterol | 4 | 3.2 |
| LDL cholesterol | 4 | 3.2 |
| Triglycerides (TGS) | 4 | 3.2 |
| Body mass index (BMI) | 10 | 8 |


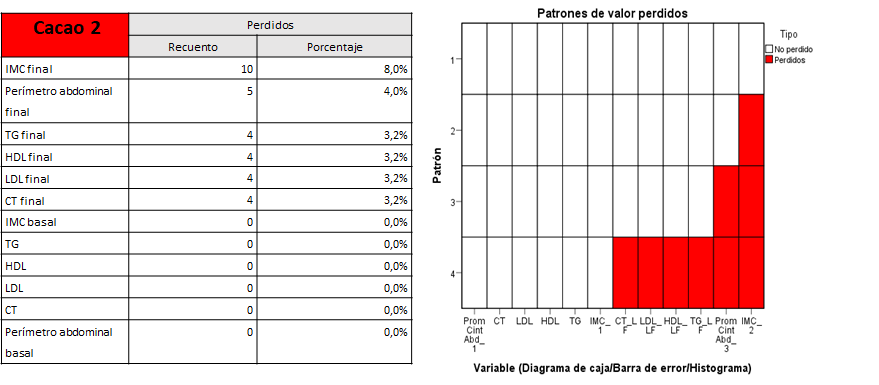


PATTERN

No missing

Missing

BMI

TGS

HDL-C

LDL-C

CT

ABD. WAIST


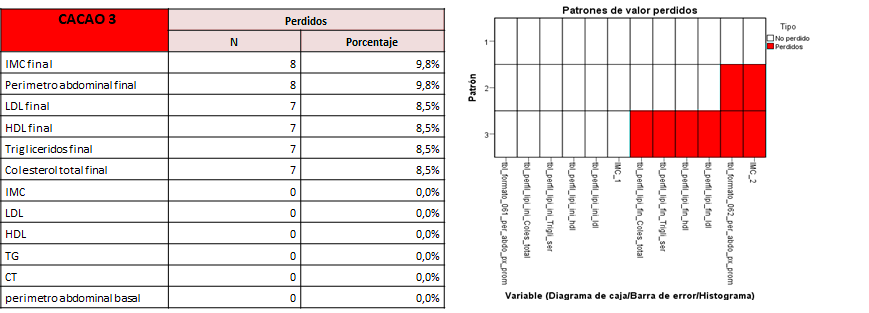


Missing

No missing

CT

TGS

HDL-C

LDL-C

ABD. WAIST

BMI

PATTERN

| **CACAO 3** | **Missing values** | |
| --- | --- | --- |
|  | **N** | **%** |
| Abdominal waist | 8 | 9.8 |
| Total cholesterol | 7 | 8.5 |
| HDL cholesterol | 7 | 8.5 |
| LDL cholesterol | 7 | 8.5 |
| Triglycerides (TGS) | 7 | 8.5 |
| Body mass index (BMI) | 8 | 9.8 |

**Annex 3.** Graphical analysis of publication bias

Funnel plot total cholesterol

Funnel plot LDL cholesterol

**
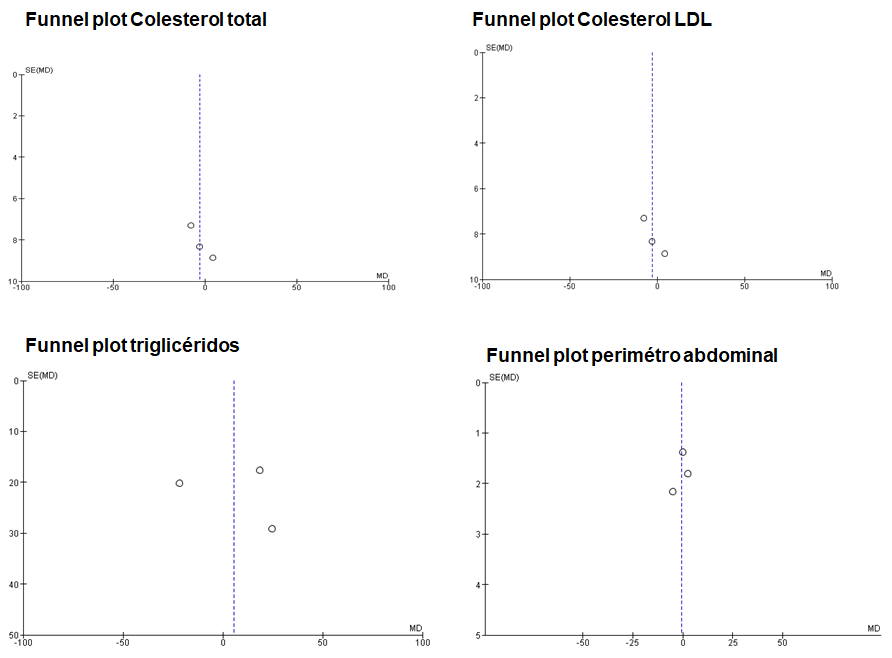

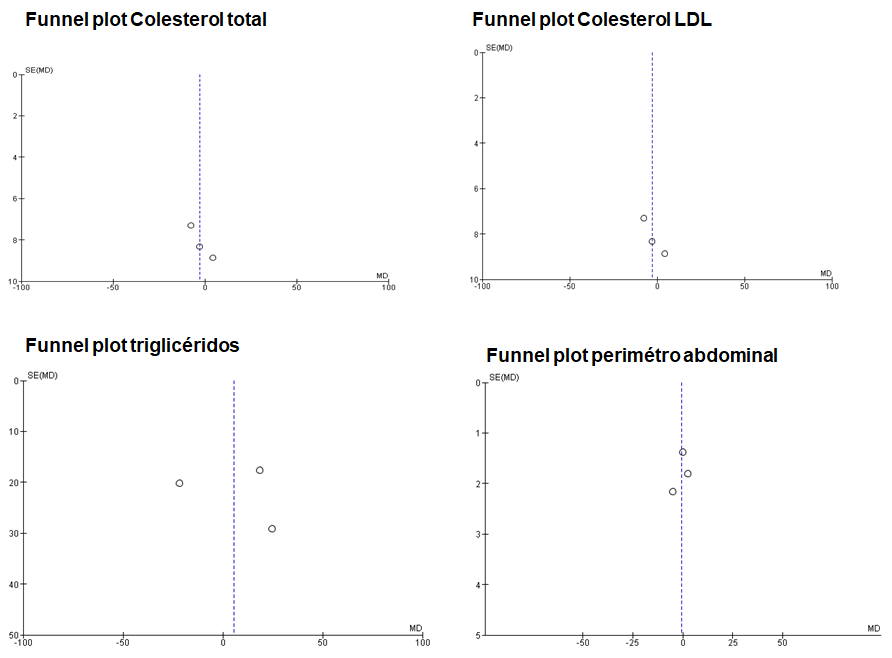
**

Funnel plot Triglycerides

Funnel plot abdominal waist

**Annex 4.** GRADE table

| Certainty assessment | | | | | | | № of patients | | Effect | Certainty | Importance |
| --- | --- | --- | --- | --- | --- | --- | --- | --- | --- | --- | --- |
| № of studies | Study design | Risk of bias | Inconsistency | Indirectness evidence | Imprecision | Other considerations | Dark chocolate | placebo | Absolut (95% CI) |  |  |
| **LDL-C** | | | | | | | | | | | |
| 3 | Randomized trials | It's not serious | It's not serious | Serious^e^ | serious ^a^ | publication bias was strongly suspected all possible residual confounders could reduce the demonstrated effect ^b^ | 101 | 174 | MD **2.79**  (-11.93; 6.35) | ⨁⨁◯◯ Low | IMPORTANT |
| **HDL-C** | | | | | | | | | | | |
| 3 | Randomized trials | It's not serious | serious ^c^ | Serious^e^ | serious ^a^ | publication bias was strongly suspected all possible residual confounders could reduce the demonstrated effect ^b^ | 101 | 174 | MD **0.55**  (-4.27 ;3.16) | ⨁◯◯◯ Very low | IMPORTANT |
| **TC** | | | | | | | | | | | |
| 3 | Randomized trials | It's not serious | serious ^c^ | Serious^e^ | serious ^a^ | publication bias was strongly suspected all possible residual confounders could reduce the demonstrated effect ^b^ | 101 | 174 | MD **1.79**  (-16.6 ;13.03) | ⨁⨁◯◯ Low | IMPORTANT |
| A**bdominal waist** | | | | | | | | | | | |
| 3 | Randomized trials | It's not serious | serious ^d^ | Serious^e^ | serious ^a^ | publication bias was strongly suspected all possible residual confounders could reduce the demonstrated effect ^b^ | 101 | 174 | MD **0.59**  (-4.29 ;3.1) | ⨁⨁◯◯ Low | IMPORTANT |
| **Triglycerides** | | | | | | | | | | | |
| 3 | Randomized trials | It's not serious | It's not serious | Serious^e^ | serious ^a^ | publication bias was strongly suspected all possible residual confounders could reduce the demonstrated effect ^b^ | 101 | 175 | MD **5.36** (23.3 ;34.02) | ⨁⨁⨁◯ Moderate | IMPORTANT |

CI: Confidence interval; MD: Mean difference

Explanations

1. Wide confidence intervals with small sample.
2. Very few studies and no systematic review
3. Heterogeneity is moderate. However, the results are not very different from those seen in other studies.
4. Heterogeneity is high. However, the results are not very different from those seen in other studies
5. Surrogate outcome.
